# The Swedish Fracture Register – ten years of experience and 600,000 fractures collected in a National Quality Register

**DOI:** 10.1186/s12891-022-05062-w

**Published:** 2022-02-11

**Authors:** Michael Möller, Olof Wolf, Carl Bergdahl, Sebastian Mukka, Emilia Möller Rydberg, Nils P. Hailer, Jan Ekelund, David Wennergren

**Affiliations:** 1 Institute of Clinical Sciences, Sahlgrenska Academy, University of Gothenburg, Gothenburg, Sweden; 2 Department of Orthopaedics, Sahlgrenska University Hospital, Gothenburg/Mölndal, Sweden; 3 Department of Surgical Sciences, Orthopaedics, Uppsala University, Uppsala, Sweden; 4 Department of Surgical and Perioperative Science (Orthopaedics), Umeå University, Umeå, Sweden; 5 Centre of Registers Västra Götaland, Gothenburg, Sweden

**Keywords:** National quality register, Fracture, Classification, Fracture outcome

## Abstract

**Background:**

Before the creation of the Swedish Fracture Register (SFR), there was no national quality register that prospectively collects data regarding all types of fractures regardless of treatment in an emergency setting. Observational data on fractures registered in a sustainable way may provide invaluable tools for quality improvements in health care and research.

**Description:**

Ten years after its implementation, the Swedish Fracture Register has 100% coverage among orthopaedic and trauma departments in Sweden. The completeness of registrations reached in 2020 69–96% for hip fractures at the different departments, with the majority reporting a completeness above 85%. The Swedish Fracture Register is a fully web-based national quality register created and run by orthopaedic professionals, with financial support from public healthcare providers and the government. All users have full access to both the registration platform and all aggregated statistics in real time. The web-based platform was created for use in health quality registers and it has easily gained acceptance among users. The register has gradually developed by the addition of more fracture types and skeletal parts.

Research activity is high and 31 scientific publications have been published since 2016. The strategy from the start was to publish validation data and basic epidemiological data. However, over the past few years, publications on outcomes, such as re-operations and mortality, have been published and four register-based, randomised, controlled trials are ongoing.

**Conclusion:**

It is possible to create a fracture register, to gain professional acceptance and to collect fracture data in a sustainable way on a national level if the platform is easy to use. Such a platform can also be used as a randomisation platform for prospective studies.

## Background

Injuries to the skeletal system are a major cause of disability in all age groups and the treatment of fractures and their sequelae constitutes a major part of total societal spending on health care [1]. Thousands of studies have addressed cohorts of fracture patients, but, to the best of our knowledge, the Swedish Fracture Register (SFR) was the first population-based register designed for prospective data collection on patients with all types of fractures, regardless of treatment [2]. Ten years after its initiation in 2011, no other quality register of this kind has been constructed, implemented and used for quality improvement and research on fracture treatment in all body parts.

The SFR has evolved from being a single-centre register with data collection on only two fracture types in 2011 to include all fracture types and reach 100% national coverage in 2021: All 54 trauma and orthopaedics departments in Sweden participate in data entry in the SFR. The SFR became the first platform for register-based, randomised, controlled trials (r-RCTs) in fracture care [3, 4]. The complexity of registrations and the need for registrations to be made within the setting of accident and emergency departments are obstacles to overcome. Legal issues in many countries limit the way registers can be constructed and used. The personal identity number (PIN) given to every Swedish citizen at birth or after permanent immigration plays a key role in the way quality registers can be maintained and cross-linked. Internationally, there are many local or regional databases that are mainly run by hospitals or groups of hospitals [5, 6]. The early development of the SFR has been described previously [2, 7], and the aim of the present study is to describe and discuss the further development of the SFR as a tool for research and quality improvement.

### Construction and content

In the SFR, information on fractures of all types is collected, except for those to the ribs and skull, which are not treated by orthopaedic surgeons in Sweden. All data entry is performed by the orthopaedic resident or surgeon. Fractures in all locations of the adult skeleton (with closed physes) are registered. Fracture registration in children (with open physes) is limited to long bone fractures, mainly because the inclusion criterion is a positive radiographic finding. In small children in particular, many injuries to fingers, toes and clavicles are diagnosed and treated without radiographic confirmation.

Following a brief section on the trauma mechanism, the fracture/−s are classified and subsequently used as the starting point for treatment registrations. A time line of treatments with planned treatments or re-operations always follows the specific registered fracture, even in a polytrauma situation.

In Table 1 a time-line for developments in the Fracture Register is shown (Table 1).

**Table 1:** Development of features in the Swedish Fracture Register

When?	What?
2010	Creation of the database platform
2011	Registrations; humeral and tibial fractures
2011	Patient-reported outcomes on paper forms
2012	Registrations; all fracture types in adults except in the spine
2015	Registrations; fractures in children
2015	Registrations; fractures in the spine
2015	Registrations; periprosthetic fractures
2016	Registrations visible at departments not performing the initial registration
2019	Automatic information exchange fracture vs arthroplasty register
2019	EQ5D-3 L replaced by EQ5D-5 L
2019	Patient-reported outcomes on electronic forms
2020	Knowledge support system for ankle fractures

The main feature of a well-functioning quality register is easy data input and readily available data for extraction and analysis. In the SFR, three outcome measurements are available; re-operations, patient-reported outcome measurements (PROM) and mortality.

First, the reoperation rate is dependent on accurate registrations of performed re-operations. This process, with its strengths and limitations, has been validated in defined cohorts [8, 9]. 

The second outcome is PROM, consisting of the generic EQ5D-5 L and the Short Musculoskeletal Function Assessment (SMFA). PROM registration went digital in 2019 when all patients were invited to answer the EQ5D-5 L and the SMFA questionnaires electronically. A few weeks after the injury a code is sent by standard mail to all patients, asking them to answer the web-based questionnaires using a recall technique on their functional status the week before the fracture occurred. Those who respond are invited to fill out the one-year follow-up questionnaires as well. The response rate is monitored continuously on the website at unit level and ranges from approximately 65% as its best at injury time and 15% as the lowest at 1 year, depending on age group, fracture type and department. Studies evaluating PROM results and response rates in the SFR are ongoing, but only a few have so far been published [10, 11].

Mortality rates are available as the third outcome measurement [12]. Death dates from the Swedish Tax Agency are updated on a daily basis to the SFR, enabling calculations of mortality rates.

### Database improvements

A quality register needs to be distinguished from a research database. A quality register has to be sustainable, using only a minimum of variables, and a user-friendly interface for registration. Otherwise, if too many variables are included, as they are in a research database, there is a risk that no registrations whatsoever will be made in busy working hours.

The SFR is fully web based and more than 3000 users can access the website to register fractures. At the same website, information is available in the form of aggregated data from the SFR and also regarding detailed data for every single fracture registered at the user’s own department. A great deal of effort has been put into involving the user through the availability of data and also recently by using data as knowledge support.

Communication from a register to its users is essential when running a quality register based on non-mandatory registrations. Published recurring newsletters, half-yearly reports specific to each department and a frequently updated website are part of the strategy. The annual report is published with data and analyses and scientific summaries of different fracture locations [13–21]. On the website, real-time data on performance appear and the number of patients included in ongoing r-RCTs is shown. All users have full access to statistics on an aggregated level. This enables the very important notion among users that “we own the data that we have entered and we can easily access and use them”.

### Specific feature for secondary fracture prevention

One register feature that has real potential to improve clinical practice is the search engine developed in the SFR to find fractures related to osteoporosis. As a database feature, it is very simple, but it has the potential to resolve an otherwise almost insoluble task. Many patients have fragility fractures, some of which also suffer from osteoporosis [22–26]. Fragility fractures are a growing concern due to their increasing incidence and they are associated with huge costs to society [1]. Secondary prevention with advice, i.e. fracture liaison services, change of lifestyle factors and medication with antiresorptive drugs, significantly reduce the rate of new fractures after the first fragility fracture [27]. However identify patients with recently sustained fragility fractures have previously been difficult. Using the database feature developed for the SFR, the task of finding these patients can now be performed easily and automatically. If the fracture has been registered in the SFR, the register provides users with a feature allowing for a standardised search for suspected osteoporosis-related fractures. All fractures to the upper arm, wrist, spine, pelvis or femur in patients 50 year of age or above can be retrieved with PINs. These patients can subsequently be offered further investigations of bone mineral density and antiresorptive treatment, if necessary. This feature is used by many departments active in the SFR and the time saving adds up to one of the easy pay-backs after implementing the SFR. In fact this search option will be the foundation of the ongoing development of regional and national guidelines for secondary fracture prevention in Sweden.

### User interface and value

The interface in the SFR is user friendly, and the use of pictograms for classification is the essential starting point. The alternatives for treatment registrations which are then available are only those relevant to the chosen fracture. This makes registrations more accurate and less time consuming. Even though data entry is considered fast and easy, it is probably beneficial to adherence to registration if more features which will benefit the surgeon making the registration are added. The SFR can be regarded as an education tool that teaches the young surgeon how to classify fractures. The use of statistics can teach the relationship between the severity of the fracture, choice of treatment and outcome. If feedback is given at the time of fracture classification and/or at the time of treatment registration, this can benefit both the young surgeon and the quality of registration, as well as the standard of care.

### Real-time feedback

In 2020, a system for real-time feedback regarding ankle fractures was implemented in the SFR. After a pilot project had been evaluated, the system was introduced nationwide. It aims at ensuring that the chosen classification takes account of both radiographic findings and clinical examination results. The evident background is the importance of ankle joint congruity and the observed difficulty involved in correctly classifying ankle fractures [11]. An error in fracture classification could be the starting point of incorrect treatment. The feedback function has obtained a high degree of acceptance, but its influence on decision-making is still to be evaluated.

### Coverage

The implementation process has been described for the early years [7] and in 2020, all 54 orthopaedic departments treating fractures in Sweden had committed to register fractures in the SFR on a daily basis (Table 2). Other national fracture registers have been planned, but to date none has been implemented in daily practice. In recent years, some more elaborate plans have been published [28].

**Table 2:** Time line for achievements in the Swedish Fracture Register

When?	What?
2011	1/54 departments participating
2012	6/54 departments participating
2013	16/54 departments participating
2014	27/54 departments participating
2015	36/54 departments participating
2015	First scientific publication
2015	100,000 fractures registered
2017	45/54 departments participating
2019	47/54 departments participating
2019	First orthopaedic r-RCT
2019	First thesis based on SFR data
2020	Annual registration over 100,000 fractures
2020	500,000 fractures registered
2020	54/54 departments participating = 100% coverage
2021	30th scientific publication
2021	600,000 fractures registered

### Completeness

Completeness of registrations in a national quality register is essential, if a high degree of external validity is to be obtained. The continuous assessment of completeness is needed and this requires a reference with which the data in the SFR can be compared. The reference data must be easy to retrieve and must contain the “true” number of fractures treated in the country. The only source of data that may fulfil these criteria in Sweden is the National Patient Register (NPR). However, the matching of fractures in the SFR with the NPR is not without its challenges. Since the NPR overestimates the true number of fractures an algorithm for case selection in the NPR has been developed [29]. This enables annual completeness analyses of fracture registrations in the SFR using the NPR for comparison. Since 2017, annual analyses of completeness based on this algorithm have been published on the SFR website, at the Swedish Board of Health and Welfare and in the annual reports from the SFR. Fractures to the humerus, wrist, hip, femur, tibia and ankle in adults and humeral and femoral fractures in children are evaluated. Most departments achieve completeness according to the current algorithm in the area of 70–90% for the evaluated fracture types [13–21]. In 2020 hip and femur fracture registrations ranged from 69 to 96% (mean approximately 85%) and wrist fracture registrations from 31 to 96% (mean approximately 70%) among the 47 departments registering fractures during the entire 2020.

### Research activity

Since the SFR is a new national quality register and the first of its kind, there was a need to evaluate the validity of data collected in the SFR. The first studies to be conducted were therefore validity studies regarding the accuracy of fracture classification in the SFR, completeness in fracture registration and evaluation of responders versus non-responders regarding PROM [11, 29–35]. The next reasonable step was to conduct descriptive studies of the epidemiology, incidence and mortality of different fractures [22–26, 36–40]. Studies designed to evaluate outcome after the treatment of fractures have been conducted and many are ongoing [8, 9, 41, 42]. Most publications have focused on the validation of essential features in the SFR or have been descriptive, basic epidemiological studies, as was the intention described above. For details on the development, see Table 2.

One of the main developments of the SFR database functionality in recent years is the creation of a research study platform for r-RCTs. Two large r-RCTs have been started and are ongoing since 2019 and 2020 respectively. The protocols for the Hipsther and the Duality studies have been published [3, 4]. Both studies focus on hip fractures and follow small studies in the same field. The aim is to use the national quality register platform to enable many departments to co-operate and include 1400–1600 patients per study over a study period of approximately 4 years. To the best of our knowledge, the Hipsther study was the first r-RCT in orthopaedics. A study on the treatment of spine fractures, Sun Burst, started in 2021 and two more studies will start in 2022. The FFT (fracture Fragility Trial) will investigate secondary fracture prevention. The recruitment of patients will use the SFR database to search for recently sustained fragility fractures. Following consent to participate, patients will be randomised to either a bone-modifying drug or a placebo in a double-blinded way. In this way, the non-mandatory registration made by professionals has resolved a highly complex logistical problem that involves the same category of patients and also serves as the foundation of a r-RCT. A decline in fracture incidence following widespread and successful anti-osteoporotic treatment is likely to occur. A change in incidence of this kind will be possible to monitor in the SFR, since time trends in fracture occurrence are continuously analysed. The Daicy-study is a cluster randomised trial that will investigate the rate of deep infection after hemiarthroplasty for displaced cervical hip fractures in over 7000 patients randomised between single and dubble antibiotic impregnated bone cement.

In these r-RCTs, the database is used not only as a tool to find the patients for an ongoing study but also as the screening instrument. In the first step, the registering surgeon at sites active within the study will be advised that the patient appears to fulfil the inclusion criteria for the specific study. In the second step, the screening process can take place on the research study platform and, after a four-question screening including informed consent, the patients, if eligible, can be randomised to different treatment methods. In this way, it is safe and easy to include patients with an acute fracture and also to manage randomisation without delaying the time to surgery. The data from screening and onwards are kept as a separate study database, while treatment is registered in the SFR as usual. All follow-up including outcomes in terms of mortality, re-operation and dislocation respectively, will take place by linking with other registers. Half the orthopaedic departments (from small local hospitals to university hospitals) in Sweden participate in these studies, thereby enabling a rapid inclusion of patients.

### Descriptive data

**Fig. 1:**
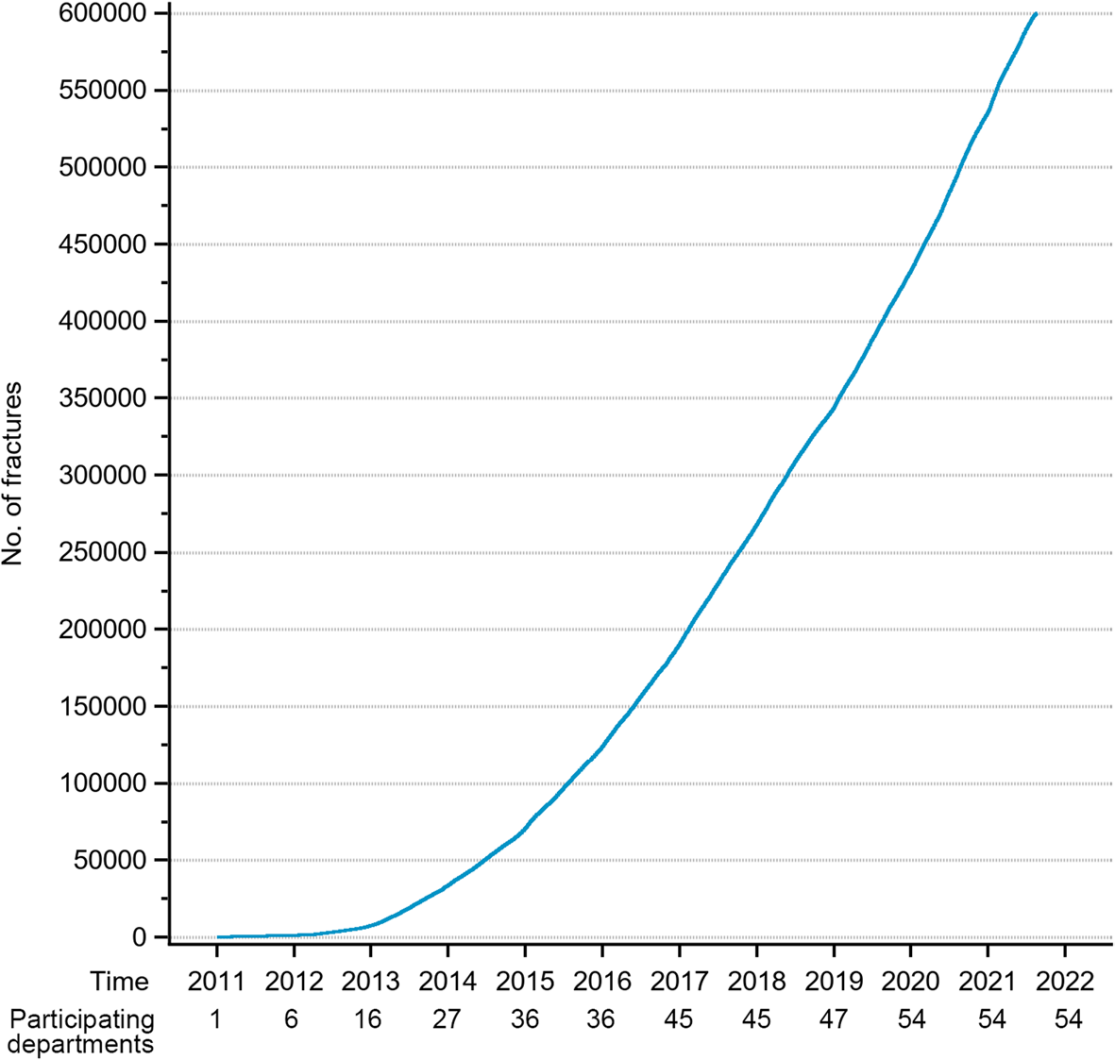
Cumulative number of registered fractures and participating departments 2011–2021

**Fig. 2:**
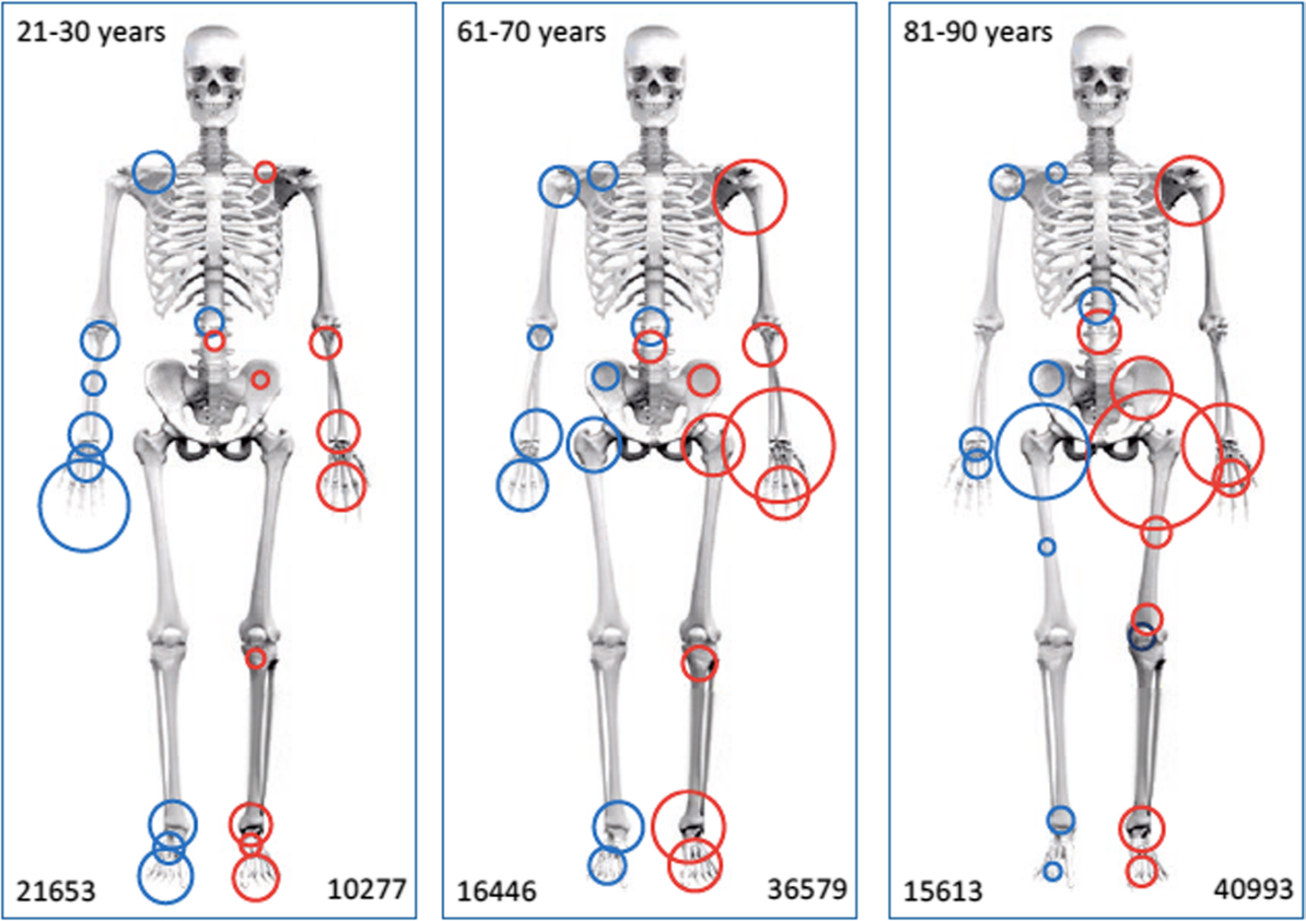
**a-c** The ten most frequent fracture locations are shown with circle sizes proportional to the number of registered fractures in age groups **a** 20–30 years, **b** 60–70 years and **c** 80–90 years in blue for men and red for women. Number of fractures are shown at the bottom of the figures

**Fig. 3:**
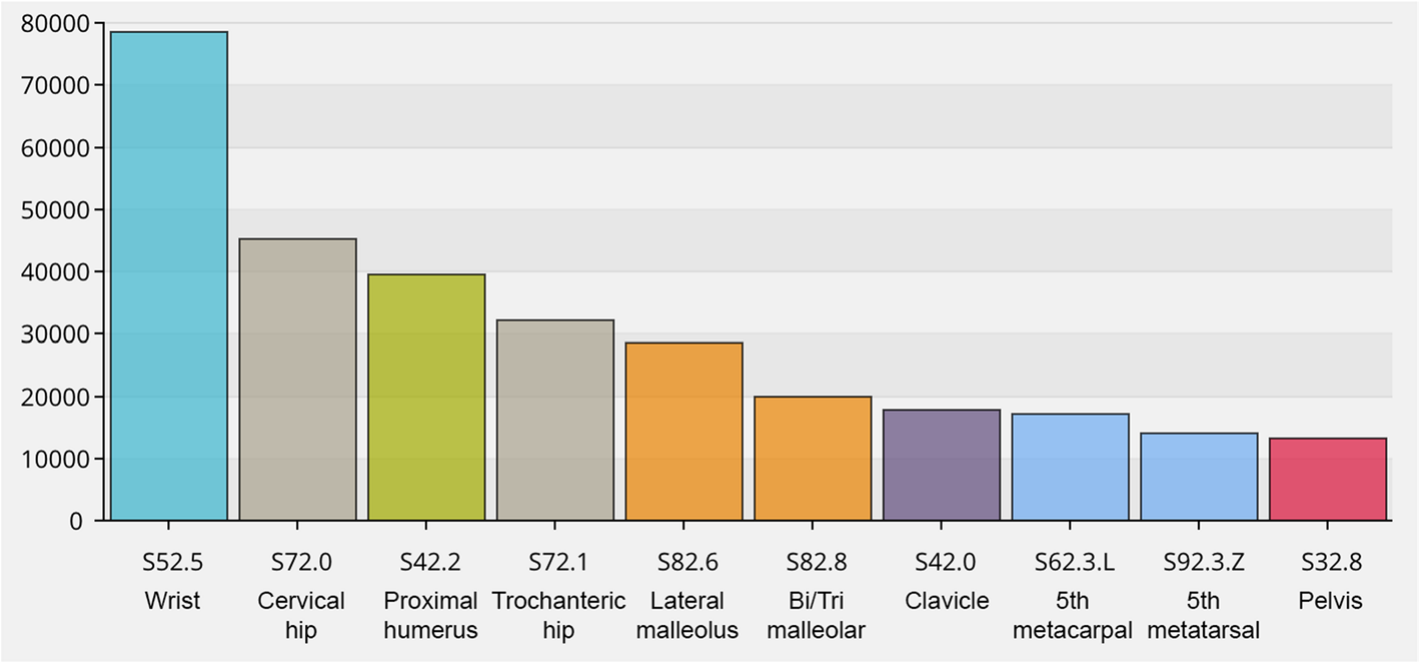
The ten most common fracture types in adults (fractures with closed physes) 2012–2021 (*n* = 306,000)

**Fig. 4:**
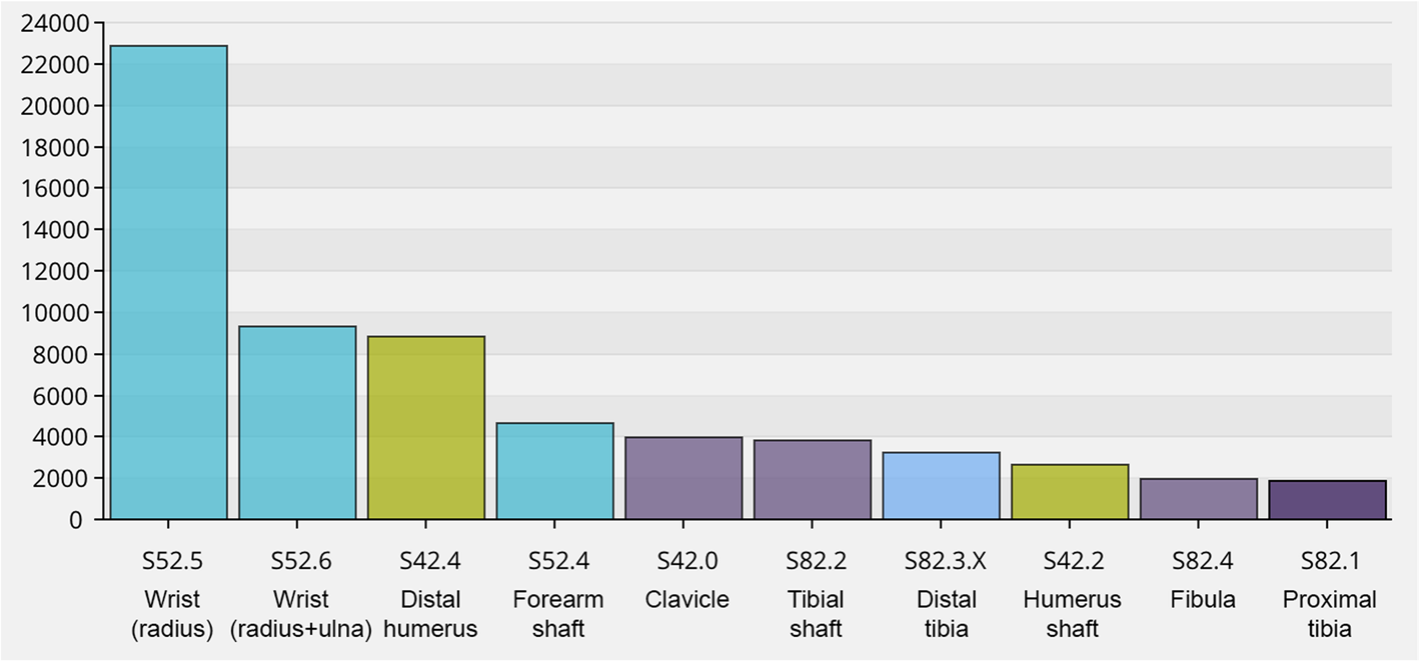
The ten most common fracture types in children (fractures with open physes) 2015–2021 (*n* = 63,227)

**Fig. 5:**
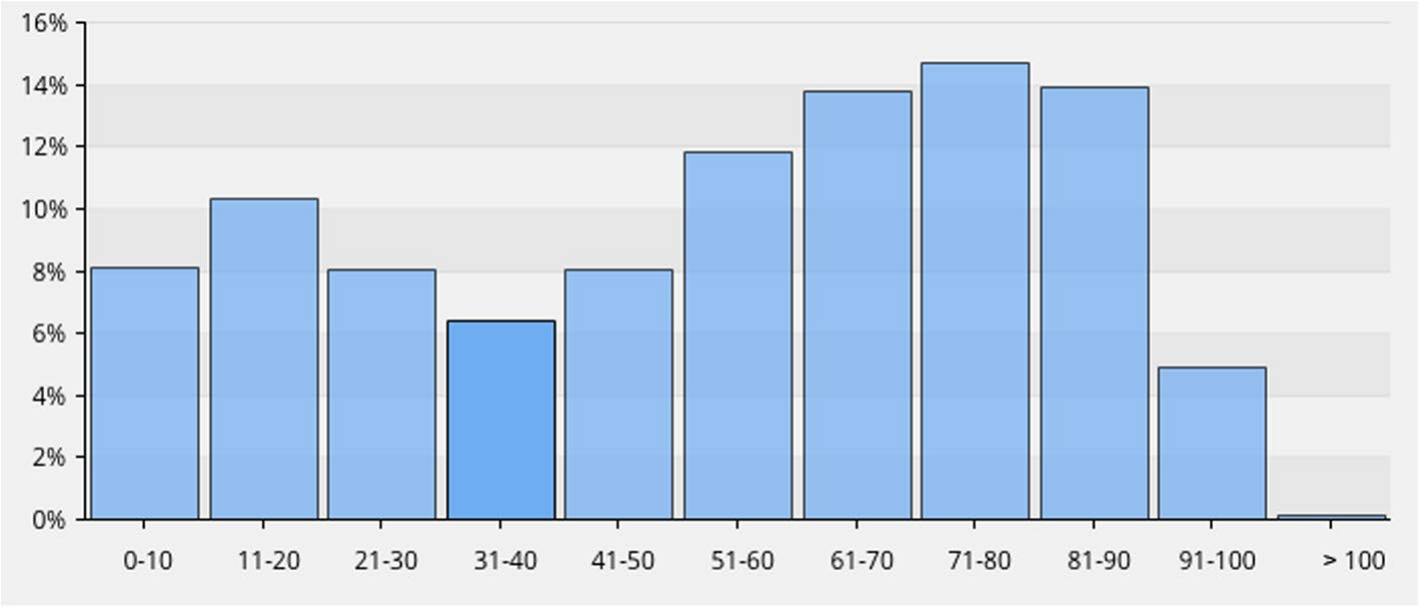
Distribution in age groups of registered individuals with fractures 2011–2021 (*n* = 604,245)

As an illustration of how users and healthcare providers are able retrieve aggregated data, the following section contains some examples of the statistics available from the database. In 2020, 103,400 fractures were registered, and in August 2021, the total number of registered fractures reached 600,000 (Fig. 1). The 10 most frequent fracture locations are shown in Fig. 2 for three defined age groups (a, b and c). In overall terms, the most common fracture types for both genders taken together, in adults and children, are shown in Figs. 3 and 4. In Fig. 5, the distribution of age at fracture is shown for all registered fractures, demonstrating that the most common age group sustaining a fracture is the group of individuals aged between 71 and 80 years.

### Future perspectives

The future perspectives include the use of more appropriate and diversified PROMs. The implementation in 2019 of web-based questionnaires created an opportunity to use different PROMs for different fracture locations and also using fewer questions. In this area, the SFR is confined to following the ongoing development of available means of collecting PROMs developed by the Swedish Association of Local Authorities and Regions (SALAR).

The implementation of the Medical Device Regulation in all European countries from May 2021 imposes significant obligations on both implant manufacturers and caregivers relating to the documentation of implant use, traceability and post-market surveillance. The SFR has the potential to provide detailed data on treatment success related to different implants. A recently started project aims to implement the inclusion of implant data using bar-code readers and the GTIN specification made available by the implant producers to the SFR. If this feature is implemented successfully, data on implant performance can be used to meet the legal traceability requirements in Europe. The introduction of new implants can be surveilled in detail with prospectively collected data. This can ensure the step-wise introduction of new implants with the potential for the early detection of less favourable implant designs on the market.

A 100% coverage among fracture-treating orthopaedic and trauma departments was achieved early in 2021. The next step in the development is the inclusion of the six university hospital hand surgery departments. In Sweden, only a small number of fractures are treated at private units. In the future, data from these units could contribute data, in particular data on minor re-operations, such as the removal of metalware.

## Conclusions

The creation of a user-friendly National Quality Register, like the unique Swedish Fracture Register, has enabled registrations of fractures on national scale. Real time registrations have made pragmatic rRCTs possible for patients with hip fractures. A high degree of coverage and completeness has been obtained due to gradual improvements and close co-operation with the orthopaedic profession. Future developments should focus on the addition of features that can increase the utility of data and the value to users making their registrations during busy working hours.

## Data Availability

The datasets generated and/or analysed during the current study are available at the SFR (www.frakturregistret.se) with a log-in which can be obtained from the corresponding author in response to a reasonable request.

## Abbreviations

SFR: Swedish Fracture Register
NPR: National Patient Register
SMF: Short Musculoskeletal Function Assessment
SALAR: Swedish Association of Municipalities and Regions
STA: Swedish Tax Agency
